# The effect of vitamin D status on the occurrence of Kawasaki Disease: a meta-analysis

**DOI:** 10.1186/s12887-024-04768-1

**Published:** 2024-04-29

**Authors:** Haixiang Zhang, Fuyong Jiao, Jiaojiao Wang, Cuixiang Xu, Kejin Zhang

**Affiliations:** 1 Shaanxi Provincial Key Laboratory of Infection and Immune Diseases, Shaanxi Provincial People’s Hospital, 256# Youyi West Road, Xi’an, Shaanxi, 710068 China; 2 Shaanxi Engineering Research Center of Cell Immunology, Shaanxi Provincial People’s Hospital, Xi’an, 710068 China; 3 Shaanxi Provincial Kawasaki Disease Treatment Center/Children’s Hospital of Shaanxi Provincial People’s Hospital, Xi’an, 710068 China; 4 Department of Medicine, School of Medicine, Yan’an University, Yan’an, 716000 China; 5 Department of Biological Sciences, College of Life Science, Institute of Population and Health, Northwest University, Xi’an, 710069 China

**Keywords:** Kawasaki disease, Vitamin D, 25-hydroxyvitamin D, Coronary artery lesion

## Abstract

**Aim:**

The relationship between vitamin D status and Kawasaki Disease (KD), as well as coronary artery lesion (CAL), has yet to be established.

**Methods:**

A meta-analysis was conducted to assess the correlation between vitamin D status and KD, as well as the impact of vitamin D status on the progression of KD into CAL.

**Results:**

The meta-analysis revealed a consistent and significant association between serum 25(OH)D level and the occurrence KD (studies *N* = 22; *z* = -3.51, *P* < 0.001). Patients with KD had markedly lower levels of vitamin D than healthy controls (*SMD*: -1.30 ng/mL, 95%CI: -2.05 to -0.55 ng/mL).

**Conclusion:**

The study provided evidence supporting a significant association between lower serum vitamin D levels and the occurrence of KD, particularly within the Chinese population. However, the findings did not suggest a direct impact of vitamin D on the development of CAL in KD patients.

**Supplementary Information:**

The online version contains supplementary material available at 10.1186/s12887-024-04768-1.

## Introduction

Kawasaki disease (KD), also referred to as mucocutaneous lymph node syndrome, is an autoimmune disease that was initially identified by Kawasaki [1] in 1967. This pediatric disorder is characterized by moderate systemic vasculitis and predominantly affects children under the age of 5 worldwide, with Asia exhibiting the highest incidence rates. Despite continued efforts to address the condition, the incidence rate of KD is on the rise [2, 3].

Although the exact mechanism behind the onset of KD remains unclear, most researchers believe that it arises from an excessive immune system response of genetically vulnerable children following infection [4]. While the symptoms of KD typically resolve spontaneously within 4—8 weeks [5], a significant number of children – ranging from 20%—40%—may suffer from heart damage [6]. This damage is predominantly due to the development of coronary artery lesions (CAL) – the primary complication associated with KD. In cases where timely treatment measures (e.g., intravenous immunoglobulin (IVIG) and aspirin treatment) are not administered, the outcome can potentially lead to death in 2% ~ 3% of patients [7]. Therefore, the development of coronary artery abnormalities is considered the leading cause of acquired heart disease in children.

Vitamin D is a group of fat-soluble secosteroids that play a crucial role in activating the innate immune system and dampening the adaptive immune system through its antibacterial, antiviral, and anti-inflammatory effects [8, 9]. The circulating 25-hydroxyvitamin D (referred to hereafter as 25(OH)D) [10], consisting of 25-hydroxyvitamin D2 (25(OH)D2, mainly via diet) and 25-hydroxyvitamin D3 (25(OH)D3, synthesized in the skin, or absorbed from an animal source), are widely used to assess an individual’s vitamin D status. In the body, 25(OH)D is converted and hydroxylated to the biologically active 1,25-hydroxyvitamin D (1,25(OH)D) in the liver and kidneys [11]. However, the form of 1,25(OH)D is quantitatively minor, unstable with a very short half-life, and is induced by the drop in ionized calcium. So, generally, the 25(OH)D test in serum is an accepted indicator of vitamin D status [12], and is widely used in studies. 25(OH)D is critical in regulating immunologic processes and plays a significant role in the pathological status of cardiovascular disease. Accordingly, the hypothesis emerged that the level of 25(OH)D could impact the development of KD in CAL.

However, the association between 25(OH)D and KD is still unestablished and even contradictory. Serological studies comparing 25(OH)D levels in patients with KD against healthy controls have reported divergent results. Twelve case–control studies [13–24] reported a significant reduction in serum 25(OH)D levels in KD patients, other studies [25–28] have shown converse outcomes or failed to obtain a significant difference [29]. The uncertainty in these results has been attributed to various factors, including small sample size, varied detection methods, and unspecific complications of individual differences (e.g., age, sex, seasonality, and the presence or absence of CAL). These findings underscore the criticality of serum vitamin D status in serum in the pathogenesis of KD in children and the need for understanding the underlying relationship between the two.

In order to arrive at a definitive conclusion, this study performed a meta-analysis to estimate the disparity in serum 25(OH)D levels between children with KD and a healthy control group. We sought to evaluate the potential impact of vitamin D levels on children with KD and examine the relationship between vitamin D status and the development of vascular abnormalities related to KD.

## Materials and methods

### Literature search

This systematic review protocol was submitted for registration to the International Prospective Register of Systematic Reviews (http://www.crd.york.ac.uk/prospero) on 11 November 2023 and published on 21 November 2023 (Registration ID: CRD42023399850). The study adheres to the PRISMA guidelines [30] and has completed its checklist. Searches were conducted in PubMed and Web of Science datasets for studies in English exploring the relationship between vitamin D status and KD. The search terms used were (Kawasaki disease OR Kawasaki-like syndrome OR Kawasaki syndrome OR mucoskin lymphnode syndrome) AND (vitamin D OR 25(OH)D OR 25(OH)D3 OR 25-hydroxyvitamin D3). A similar search was also conducted in China National Knowledge Infrastructure (CNKI) and Wanfang datasets using the terms “Vitamin D” and “Kawasaki Disease” for relevant reports in Chinese. Only studies published until February 2023 were included in this study, and any disagreements were resolved through discussion until consensus was reached or with the input of a third author.

The studies included in this analysis should satisfy the following eligibility criteria: (i) there must be two types of participants included, based on the diagnosis guidelines from the American Academy of Pediatrics and Cardiology Society [4], the Kawasaki Disease Research Committee in Japan in 2002 [31], and Editorial Committee of Chinese Journal of Pediatrics [32]: those who were diagnosed as KD patients with or without coronary artery lesions (CAL), and healthy individuals who are matched for age and sex; (ii) data on the mean level of 25(OH)D along with its standard deviations (SD) and/or standard error (SE) must be present for both groups of KD patients and healthy controls; and (iii) studies must offer other relevant information that highlights the difference of vitamin D status between KD patients and controls. Conversely, studies irrelevant to the association between vitamin D and KD, repeated reports or meeting summaries, and studies that lack original data will not be included in this study.

### Data extraction and quality assessment

This meta-analysis involved two independent authors who extracted specific data, which included: (i) the first author, time of publication, and sample size; (ii) the mean serum vitamin D level, standard deviation, and/or standard error for both KD and control groups; (iii) ethnicity, mean age of subjects, proportion of females in the sample, and complications of participants; and (iv) other format data that could be used to calculate the effect size value. As the main complication of KD, the difference between patients with CAL and without CAL was often discussed [15, 20, 26], and patients were categorized into three groups—KD patients with CAL (CAL group), KD patients without CAL (NCAL group), and mixed group—and were used in subsequent subgroup analysis to determine the influence of patient complication on the relationship between vitamin D status and KD. When recording the concentration value of serum 25(OH)D, the data of different units were converted and unified as ng/mL. Additionally, the quality of all articles was evaluated by two authors, in accordance with the Newcastle–Ottawa Scale (NOS) assessment scale [33].

### Statistical analysis

Two R packages, *meta* and *metafor*, were employed to conduct meta-analysis [34, 35]. The effect size of each study was measured using standard mean difference (SMD) and odds ratio (OR) effect values, alongside their 95% confidence intervals (95% CIs), to compare KD and control groups. As per Cuijpers’ recommendation [36], the more conservative random-effects model was used, which assumes that all studies stem from “multiple populations” and the true effect is normally distributed. To assess heterogeneity among the included studies, both Cochran’s Q-statistic and I 2 statistics were employed.

Studies with extreme effect sizes, also known as outliers, may raise concerns and distort the overall results of a meta-analysis [35]. To address this issue, an R program called *influence.analysis* was utilized. This program detects and removes outlier(s) among all eligible studies using various influence measures (e.g., DIFFITS, Cook’s distance, covariance ratio, etc.). After removing the outlier(s), the meta-analysis was conducted again. To account for the significant heterogeneity (*P*_het_ ≤ 0.05 or *I* ^2^ ≥ 25%) observed, the sources of heterogeneity will be considered [37]. *Begg’s* rank correlation test and *Egger’s* weighted regression test will be conducted to assess the risk of publication bias. The results of the *trim-and-fill* test will be visualized through a funnel plot. Additionally, the *leave-one-out* function will be used to examine the sensitivity of the results. To maintain the Type I error rate, the “*permutest*” command of the *Metafor* package will be utilized to conduct a permutation test with 1,000 iterations.

In order to investigate the potential causes of the observed heterogeneity and determine their impact on the correlation between vitamin D and KD, a meta-regression analysis was conducted using a mixed-effects model (which utilized a random-effects model within subgroups and a fixed-effects model among subgroups) [38]. Additionally, the model test (*Q*_M_) and goodness of fit test (*Q*_G_) were employed to gauge the effect of moderators on the relationship between vitamin D status and KD, and to determine whether other factors may contribute to the variability in effect size [35]. This meta-regression analysis involved five types of information per sample, namely, publication date, mean age of participants, the percentage of females, race, and presence of KD complications, all of which were considered as possible covariates. All significance tests were two-tailed, with a significance threshold set at 0.05.

## Results

### Characteristics of eligible studies and samples

**Fig. 1:**
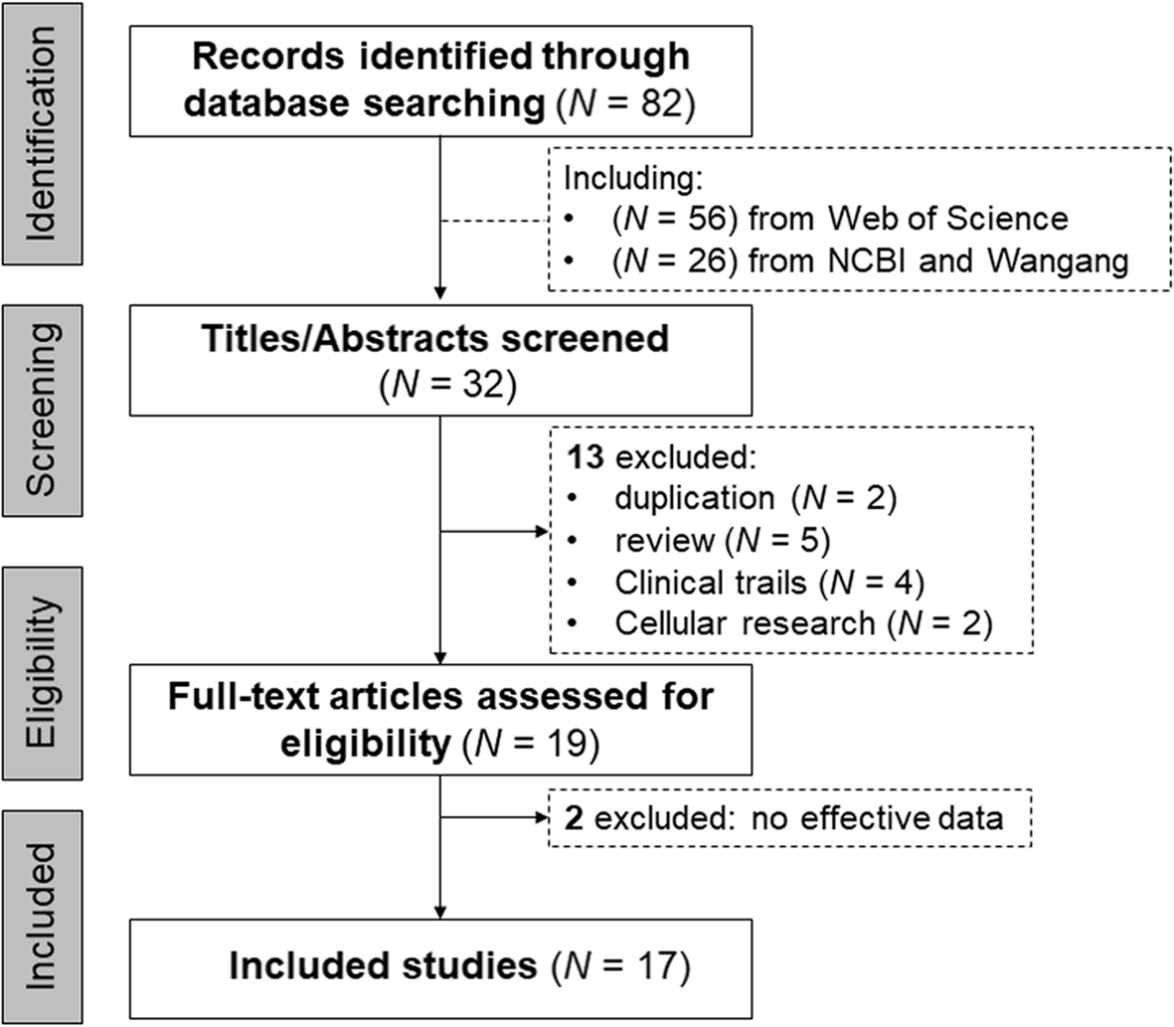
A literature reviewing for the relationship between vitamin D status and Kawasaki disease

Initially, 82 publications pertaining to the correlation between vitamin D status and KD were examined based on the inclusion criteria, as depicted in Fig. 1. Upon review of the title and abstracts, 50 studies were excluded as they were deemed irrelevant to the relationship between vitamin D and KD. Out of the remaining 32 studies, two reports were found to be duplicated, five were reviews, four were clinical trial studies, and two focused on cellular research work. After excluding two studies lacking effective data, a total of 17 eligible studies were identified and included in the ensuing meta-analysis. The eligible studies comprised 23 samples, consisting of 1,394 KD patients and 1,557 healthy controls. Of these KD patients, 272 had CAL, 423 did not suffer from CAL complications, and the remaining did not report their complications. The study included mostly Han Chinese participants (72.6%), with one sample (12.7%) from Japan [16] and two samples (14.7%) from Italy [13, 18]. This distribution is consistent with the higher incidence of KD observed in Northeast Asian countries like China, Japan, Korea, and Taiwan compared to other developed nations such as the United States and Europe. The age of the participants ranged from 0 to 5 years, with a mean age of 2.8 ± 0.8 years, and 41.9% were females. Detailed information regarding the eligible studies is provided in Table 1.

**Table 1:** The characteristics of subjects included in the meta-analysis

First author, year	Country	N	Age	Sex (%)	25(OH)D level (mean ± SD)		
					**KD**	**Control**	**unit**
**Mix patient’s studies**							
An 2016 [25]	China	45/43	3.1	46.5	69.17 ± 3.05	46.39 ± 2.22	ng/mL
Falcini 2015 [13]	Italy	60/60	1.98	30	8.9 ± 2.24	21.7 ± 4.35	mg/dL
Huang 2022 [3]	China	105/45	2.9	46.7	25.2 ± 3.65	36.1 ± 4.31	ng/mL
Okazaki 2022 [16]	Japan	86/290	2.1	42.6	17 ± 12	29 ± 15	ng/mL
Que 2022 [28]	China	98/96	1.15	31.4	62.78 ± 6.35	44.15 ± 4.65	ng/mL
Ren 2021 [17]	China	66/66	3.46	37.1	15.73 ± 3.84	46.83 ± 9.38	ng/mL
Stagi 2016 [18]	Italy	79/234	4.7	33.9	9.17 ± 4.94	23.3 ± 10.6	ng/mL
Wang 2015 [29]	China	35/25	n.a	25.7	58.12 ± 28.57	44.1 ± 30.2	ng/mL
Wen 2018 [19]	China	50/30	2.9	47.5	17.9 ± 2.45	29.76 ± 6.77	ng/mL
Zhou 2022 [23]	China	40/30	2.4	61.6	21.86 ± 7.41	35.04 ± 5.14	nmol/L
Zhu 2021 [24]	China	72/72	3.42	42.4	15.22 ± 4.47	46.37 ± 10.29	ng/mL
**CAL/NCAL patient’s studies**							
Chen 2014 [27]	China	9/30	n.a	35.4	83.9 ± 26.3	44.1 ± 30.2	ng/mL
		26/30	n.a	35.4	49.2 ± 23.8	44.1 ± 30.2	ng/mL
Chen 2019 [26]	China	30/30	3.3	42.3	93.04 ± 33.61	23.72 ± 11.05	ng/mL
		11/30	3.3	42.3	36.41 ± 28.67	23.72 ± 11.05	ng/mL
Jiang 2020 [15]	China	74/80	2.18	45.9	25.43 ± 7.24	34.23 ± 6.29	ng/mL
		24/80	2.3	37.5	18.86 ± 9.11	34.23 ± 6.29	ng/mL
Zhang 2019 [20]	China	76/58	1.8	40	33.24 ± 7.74	43 ± 13.1	nmol/L
		71/58	1.8	40	38.72 ± 10.82	43 ± 13.1	nmol/L
Zhang 2018 [21]	China	20/45	3.2	44.1	15.47 ± 4.23	39.18 ± 10.23	ng/mL
		75/45	3.2	44.1	20.93 ± 5.79	39.18 ± 10.23	ng/mL
Zhang 2016 [22]	China	63/40	3.2	38.1	15 ± 4	40 ± 10	ng/mL
		179/40	3.4	44.7	22 ± 5	40 ± 10	ng/mL

*Abbreviation*: *KD* Kawasaki disease, *CAL* KD without coronary artery lesions, *NCAL* KD without coronary artery lesions, *Mix* both CAL and NCAL included, *N* the number of cases and controls (case/control), *Sex* the proportion of female in subjects, *n.a* no applicable

### Meta-analysis

**Fig. 2:**
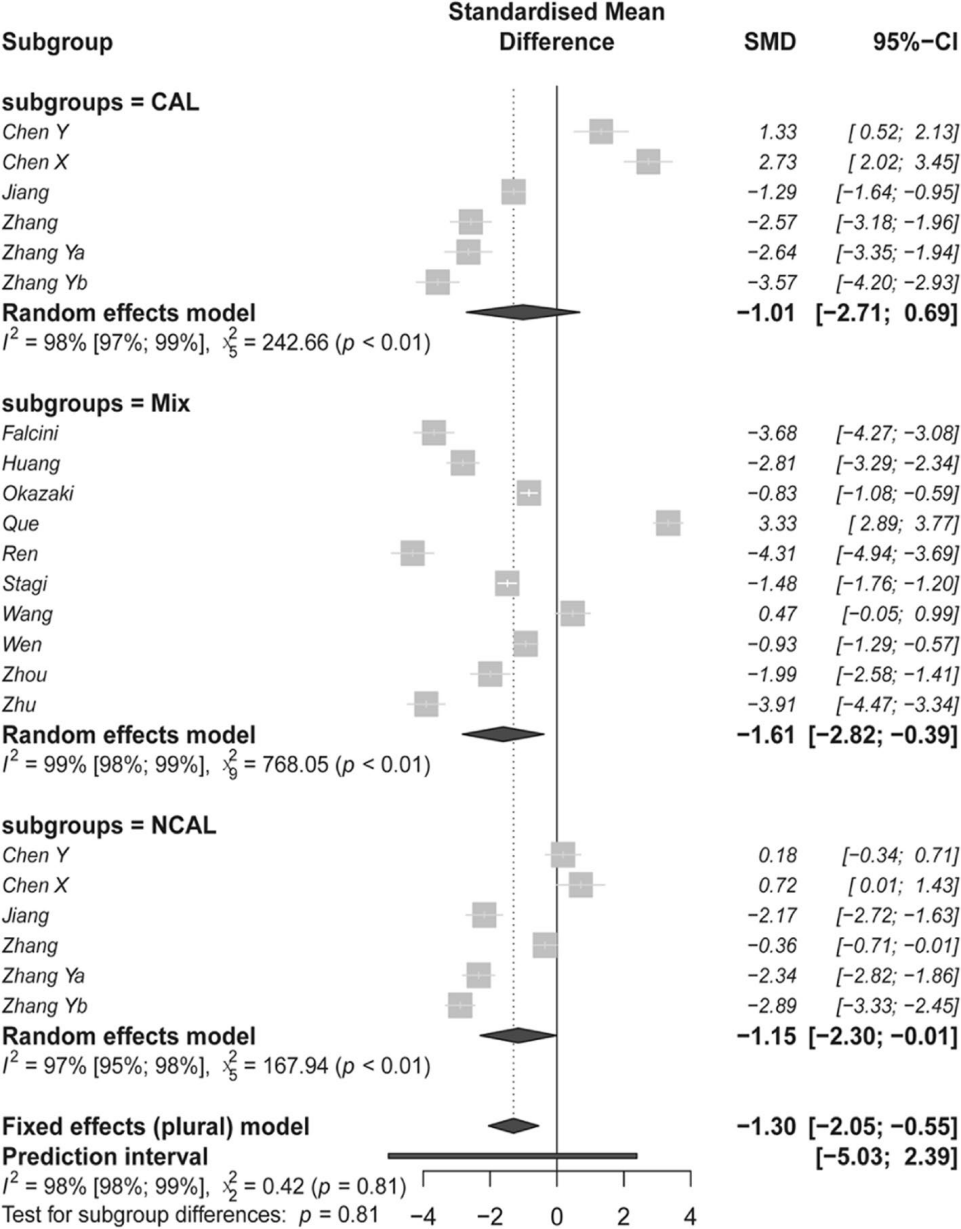
The estimated difference of serum 25(OH)D level on KD, grouped by the condition of complications. The difference of serum 25(OH)D level between patients with KD and control group was estimated and pooled through meta-analysis. Each bright gray square represented the effect value of one study. Estimates of overall and subgroups were presented in black prisms. The bright gray band below was the confidence interval of the overall effect value. SMD, standardized mean difference; CI, confidence interval; CAL, KD patients with coronary artery lesion; NCAL, KD patients without CAL

In the primary meta-analysis, Fig. 2 depicts the variance in vitamin status observed in KD patients compared to healthy control participants, with the pooled difference estimated under the random-effect model. Results indicate that patients with KD had significantly lower 25(OH)D levels than healthy controls (studies *N* = 23; *SMD* (95%CI): -0.94 (-1.72 to -0.15) ng/mL; overall effect: *z* = -2.35, P = 0.019). This corresponds to an increased risk of KD onset (*OR*: 5.50, 95%CI: 1.32 to 22.73) per 1-SD decrease in the level of vitamin D among children. The *influence.analysis* test found that the study of An et al. [25] was an outlier with low-quality data and an extreme effect size (Data S1 and Figure S1). Upon its removal, results consistently showed a significant effect of serum 25(OH)D levels on KD (studies *N* = 22; *SMD* (95%CI): -1.30 (-2.05 to -0.55) ng/mL; overall effect:* z* = -3.40, *P* < 0.001; Table 2). That was to say, for per-SD decrease in serum 25(OH)D level in children, the risk of KD increased by 11.11 (95%CI: 2.89 to 43.48) times. The permutation test with 1,000 iterations confirmed this finding (*P* = 0.007). However, a high degree of heterogeneity was still observed among these studies (Cochran’s *Q* = 1,206, *df* = 21; and *I* ^2^ = 98.26; *P* < 0.001). Sensitivity analysis showed that the result was robust, with the effect size (from -1.46 to -1.14 ng/mL) remaining within the range of the overall effect’s 95%CI interval when any single study was removed (Figure S2). Notably, neither *Begg’s* rank method (*Tau* = -0.06, *z* = 0.37, and *P* = 0.714) nor *Egger’s* linear regression test (*t* = 0.53, *df* = 20, and *P* = 0.603) detected significant publication bias in this meta-analysis.

**Table 2:** Subgroup meta-analysis by countries and condition of complication of patients

Groups	Studies	*SMD*	*SE*	95%CI		*z*	*P*
				*Lower*	*Upper*		
Overall	22	-1.30	0.38	-2.05	-0.55	-3.40	** < 0.001**
Country	*Q* = 0.94, *df* = 1, *P* = 0.331						
Chinese	19	-1.22	0.47	-2.14	-0.31	-2.61	**0.009**
Other countries	3	-1.97	0.62	-3.18	-0.76	-3.19	**0.001**
Complications	*Q* = 0.42, *df* = 2, *P* = 0.811						
Mix	10	-1.61	0.62	-2.82	-0.39	-2.59	**0.010**
NCAL	6	-1.15	0.58	-2.30	-0.01	-1.97	**0.049**
CAL	6	-1.01	0.87	-2.71	0.69	-1.16	0.247

*Abbreviation*: *SMD* standard mean difference, *SE* standard error, *KD* Kawasaki disease, *CAL* KD without coronary artery lesions, *NCAL* KD without coronary artery lesions, *Mix* both CAL and NCAL included

Further meta-analyses were conducted to compare subgroups. The studies were divided into two groups based on the nationality of the subjects: Chinese, and other countries. The results showed a consistently significant difference in serum 25(OH)D levels between children with KD and healthy controls in both groups (with Chinese samples: *N* = 19, *SMD* (95%CI): -1.22 (-2.14 to -0.31) ng/mL, overall effect: *z* = -2.61, *P* = 0.009; with other countries samples: *N* = 3, *SMD* (95%CI): -1.97 (-3.18 to -0.76) ng/mL, overall effect: *z* = -3.19, *P* = 0.001; see Table 2). However, when considering the complications of patients, inconsistent results were found between the three groups (mix; NCAL; and CAL). The significant effect size of serum 25(OH)D level was only observed in NCAL patients, mixed patients, and healthy controls (two *P*s < 0.019; see Table 2 and Fig. 2).

### Meta-regression analyses

To investigate the potential reasons for the significant heterogeneity observed in the studies included in our analysis, a meta-regression analysis was conducted. The findings revealed that the variables we examined (e.g., participant age, gender composition, publication date, race, and complications) did not contribute significantly to the observed heterogeneity (all *P* > 0.05). Furthermore, results from our model indicated that these five variables had no significant impact on the effect size of serum 25(OH)D levels on KD (*Q*_M_ = 2.89, *df* = 6, *P* = 0.82; Table S1). However, the goodness-of fit analysis showed that this model was incomplete and did not provide an accurate estimate of the true effect size of 25(OH)D on KD (*Q*_G_ = 834.96, *df* = 12, *P* < 0.01; Table S1).

## Discussion

In this meta-analysis, we confirmed the crucial impact of vitamin D on the occurrence of Kawasaki disease. Our study delved into the correlation between vitamin D status and KD while examining the impact of several pertinent factors on their relationship. Our analyses revealed a significant link between vitamin D status and KD, with children affected by KD displaying considerably lower serum 25(OH)D levels compared to healthy controls. Nevertheless, our subsequent hierarchical analysis indicated that the correlation between serum vitamin D status and KD may not be associated with the progression of CAL complications in KD patients.

A growing body of evidence from various studies demonstrates the crucial role of vitamin D in the systemic inflammatory response and the release of anti-inflammatory cytokines [39, 40]. Researchers posit that vitamin D can regulate the immune system and hence, influence the occurrence and development of KD [16]—an autoimmune disease—and its therapeutic treatment through immune system regulation [22]. This hypothesis is backed by animal, cell, and human studies which indicate that activated vitamin D (25(OH)D) plays an integral role in regulating immunologic processes [41]. It follows that individual with higher serum 25(OH)D levels may have a lower chance of developing KD [25]. This meta-analysis of seventeen published studies with approximately 1,394 KD patients and 1,527 healthy controls confirms that serum vitamin D status is significantly correlated with KD. Specifically, the serum 25(OH)D level of KD patients is significantly lower compared with that of healthy controls.

Acute systemic vasculitis, known as KD, is characterized by a range of clinical symptoms including fever, chapped lips, strawberry tongue, red eyes, and other acute systemic inflammation characteristics. However, the pathological differences that result in such broad clinical manifestations are yet to be established. Epidemiological studies have also highlighted the gender [2], age [42], and race/ethnicity-specific [43, 44] features of children with KD. The status of vitamin D can vary depending on factors such as age, sex, race, and seasonal alterations. This variability might contribute to the inconsistent findings regarding the association between serum vitamin D levels and KD observed across different case–control studies [45–48]. Moreover, the clinical manifestation heterogeneity of immune diseases linked to vitamin D metabolism further complicates the understanding of the current relationship between VD and KD. Reverse causality mentioned by Chakhtoura et al. [49] is another factor should be taken into account [50]. These factors serve as potential sources of heterogeneity that could influence the results of our analysis. Despite the extreme heterogeneity observed in all eligible studies, the meta-regression test revealed that the influence of these potential covariates was not significant enough to affect the relationship between vitamin D status and KD.

CAL is the most serious complication of KD, with about 9—20% of patients experiencing it even after routine treatment [51]. As the pathological agents for KD have not yet been determined, the development process of CAL in KD patients remains unknown [52]. Consequently, researchers have sought to understand the development process of the coronary artery and its influencing factors to intercept or reduce the risk of CAL in KD patients. Some studies have revealed a notable association between low levels of serum 25(OH)D in KD patients with CAL when compared to those without CAL and control groups [22, 27, 53]. Additionally, vitamin D supplementation and adjuvant treatment have been found to effectively mitigate the risk of CAL and improve IVIG treatment response [14]. However, seven eligible studies revealed discrepancies in their findings, with four studies indicating that the serum 25(OH)D level of KD patients with CAL was lower than that of patients without CAL [20–22, 54], and the remaining three studies revealing the opposite phenomenon [15, 26, 27] (Table 1). These inconsistent results might explain why subgroup analyses of CAL complications have not yielded consistent outcomes, signifying that the pathology of CAL in KD patients is likely multifaceted and unpredictable based solely on vitamin D levels.

The present study boasts strengths. Firstly, in addition to commonly used meta-analysis tests (such as heterogeneity, sensitivity, and publication bias analyses), the inclusion of outlier studies was detected and addressed, effectively reducing the influence of any such outlier(s); secondly, the potential impacts of other model-related covariates were also analyzed using meta-regression analysis; and lastly all estimates obtained from the meta-analysis were adjusted by permutations test. Notably, the present study employed both the random-effects model and the meta-regression method to quantitatively analyze the influence of potential variables on the relationship between vitamin D status and KD. As such, the study provides a more precise, defensible conclusion compared to previous research reports.

It is important to acknowledge the limitations of this review. Firstly, the majority of participants in the eligible studies were of Chinese ethnicity, with insufficient representation of other ethnic groups. As a result, the findings of this study may only be applicable to the relationship between vitamin D status and KD in the Chinese population, despite similar estimates found within subgroup analyses based on nationality; secondly, a surfeit immune response can theoretically give rise to a reduction in VD levels in the blood. Given that having a fever for at least 5 days is one of the criteria for diagnosing KD, the temporal gap between the occurrence of KD and the measurement of vitamin D levels, as well as the possible existence of a reverse causality [50], might disrupt our precise assessment of the relationship between the two; thirdly, it is worth noting that the activity and status of vitamin D exhibit seasonal variations [55], and the incidence of KD also demonstrates a clear seasonal pattern [56]. However, due to the limited available data, this meta-analysis cannot confirm whether seasonality is also a contributing factor to the relationship of our currently discussing. Therefore, in-depth and rigorous trials and experiments are necessary to better comprehend the potential mechanisms of vitamin D in KD incidence and development. Additionally, the impact of differences in study design, diagnostic methods, and techniques for vitamin D measurement cannot be ignored.

## Conclusion

In summary, according to current published reports, this meta-analysis study has confirmed a correlation between vitamin D levels and the occurrence of KD in Chinese population, especially. Significantly lower levels of serum 25(OH)D were found in children with KD compared to healthy controls. However, it appears that the measurement of 25(OH)D alone may not be adequate in predicting CAL occurrence in KD.

### Supplementary Information

**Supplementary Material 1.**

## Data Availability

Publicly available datasets were analyzed in this study.

## References

[1] Kawasaki T (1967). Acute febrile mucocutaneous syndrome with lymphoid involvement with specific desquamation of the fingers and toes in children. Arerugi.

[2] Holman RC (2003). Kawasaki syndrome hospitalizations in the United States, 1997 and 2000. Pediatrics.

[3] Huang GY (2022). Challenges in the diagnosis and treatment of Kawasaki disease. Zhonghua Er Ke Za Zhi.

[4] Correction to: Diagnosis, Treatment, and Long-Term Management of Kawasaki Disease: A Scientific Statement for Health Professionals From the American Heart Association. Circulation, 2019. 140(5):e181–e184. 10.1161/CIR.0000000000000703 31356128

[5] Baker AL, Newburger JW (2008). Cardiology patient pages Kawasaki disease. Circulation.

[6] Kato H, et al. Long-term consequences of Kawasaki disease. A 10- to 21-year follow-up study of 594 patients. Circulation. 1996;94(6):1379–85. 10.1161/01.cir.94.6.1379 8822996

[7] Kim DS. Kawasaki disease. Yonsei Med J. 2006;47(6):759–72. 10.3349/ymj.2006.47.6.759 PMC2687814 17191303

[8] L Bishop E (2021). Vitamin D and Immune regulation: antibacterial, antiviral, anti-inflammatory. JBMR Plus.

[9] Hewison M (2011). Vitamin D and innate and adaptive immunity. Vitam Horm.

[10] Saenger AK (2006). Quantification of serum 25-hydroxyvitamin D(2) and D(3) using HPLC-tandem mass spectrometry and examination of reference intervals for diagnosis of vitamin D deficiency. Am J Clin Pathol.

[11] Xu B (2021). Vitamin D status in children with short stature: accurate determination of serum vitamin D components using high-performance liquid chromatography-tandem mass spectrometry. Front Endocrinol (Lausanne).

[12] Holick MF (2009). Vitamin D status: measurement, interpretation, and clinical application. Ann Epidemiol.

[13] Falcini F (2015). Severe vitamin D deficiency in patients with Kawasaki disease: its possible role in the risk to develop coronary artery damage. Ann Rheum Dis.

[14] Huang J-H (2022). Value of serum 25-Hydroxyvitamin D levels in the prediction of intravenous immunoglobulin resistance in patients with complete Kawasaki disease. J Modern Lab Med.

[15] Jiang H-F, Gao J, Han W-N (2020). Study on serum vitamin D level and vitamin D receptor Fok I gene polymorphism in children with Kawasaki disease. Mat Child Health Care China.

[16] Okazaki N (2022). The impact of vitamin D on the onset and progress of Kawasaki disease. Pediatr Int.

[17] Ren J, Wu X (2021). The value of serum 25(OH)D3, NT-proBNP and IL-6 in early diagnosis of Kawasaki diaease in children. Chinese J Woman Child Health.

[18] Stagi S (2016). Severe vitamin D deficiency in patients with Kawasaki disease: a potential role in the risk to develop heart vascular abnormalities?. Clin Rheumatol.

[19] Wen H-Y, Jing X-A. Clinical significance of changes of vitamin A, 25-hydroxyvitamin D in children with Kawasaki disease. Home Medicine. 2018;7:1.

[20] Zhang X-Y (2019). Changes and clinical significance of serum 25-hydroxyvitamin D levels in children with Kawasaki disease. China Modern Med.

[21] Zhang Y (2018). The significance of changes in endogenous hydrogen sulfide and 25-hydroxyvitamin D3 levels in children with Kawasaki disease. J Imag Res Med.

[22] Zhang YD (2016). Changes in 25-hydroxyvitamin D3 level and its significance in children with Kawasaki disease. Zhongguo Dang Dai Er Ke Za Zhi.

[23] Zhou Z (2022). 1α, 25-dihydroxyvitamin D3 inhibits THP-1 cell inflammatory factor expression induced by Kawasaki disease serum through TLR4 signal pathway. Chinese J Pathophysiol.

[24] Zhu J, Peng H (2021). Serum 25 - (OH) D_ (3) Expression of NT-proBNP and IL-6 in children with Kawasaki disease and their correlation analysis. China Med Eng.

[25] An X (2016). Significance of serum 25-hydroxyvitamin D(3) and interleukin-6 levels in immunoglobulin treatment of Kawasaki disease in children. Exp Ther Med.

[26] Chen X-H (2019). Serum 25 hydroxyvitamin D3 in predicting coronary arterial lesions of Kawasaki disease. Chinese J Woman Child Health Res.

[27] Chen YL, Wang JL, Li WQ (2014). Prediction of the risk of coronary arterial lesions in Kawasaki disease by serum 25-hydroxyvitamin D3. Eur J Pediatr.

[28] Que XJ (2022). Predictive value of serum PA, NT-proBNP and 25-(OH)D3 levels on the risk of coronary artery disease in children with Kawasaki disease. J North Sichuan Med College.

[29] Wang Y-L (2015). Changes in serum 25 - (OH) D3 and T cell subsets in children with Kawasaki disease. Chinese J Woman Child Health.

[30] Moher D, et al. Preferred reporting items for systematic reviews and meta-analyses: the PRISMA statement. Ann Internal Med. 2009;151(4):264-69. 10.7326/0003-4819-151-4-200908180-00135 19622511

[31] Ayusawa M (2005). Revision of diagnostic guidelines for Kawasaki disease (the 5th revised edition). Pediatr Int.

[32] Editorial Committee of Chinese Journal of Pediatrics C.G.o.C.P.S, Immunology Group of Chinese Peediatric Society (2007). Summary of Kawasaki Disease Symposium. Chinese Journal of Pediatrics.

[33] Wells GA, D O'Connell SB, Peterson J, Welch V, Losos M, Tugwell P (2014). The Newcastle-Ottawa Scale (NOS) for Assessing the Quality of Non-Randomised Studies in Meta-Analyses. in Symposium on Systematic Reviews: Beyond the Basics.

[34] Balduzzi S, Rücker G, Schwarzer G (2019). How to perform a meta-analysis with R: a practical tutorial. Evid Based Ment Health.

[35] Viechtbauer W (2010). Conducting meta-analyses in R with the metafor package. J Stat Softw.

[36] Cuijpers P (2016). Meta-analyses in mental health research. A practical guide. Amsterdam, the Netherlands: Pim Cuijpers Uitgeverij.

[37] Higgins JP, Thompson SG (2002). Quantifying heterogeneity in a meta-analysis. Stat Med.

[38] Harrer M (2019). Doing Meta-Analysis in R: A Hand-on Guide.

[39] Dong Y (2022). Effects of vitamin D(3) and marine omega-3 fatty acids supplementation on biomarkers of systemic inflammation: 4-year findings from the VITAL randomized trial. Nutrients.

[40] Ribeiro VR (2021). Vitamin D modulates the transcription factors of T cell subsets to anti-inflammatory and regulatory profiles in preeclampsia. Int Immunopharmacol.

[41] Qi XL (2017). 1,25-Dihydroxyvitamin D3 regulates T lymphocyte proliferation through activation of P53 and inhibition of ERK1/2 signaling pathway in children with Kawasaki disease. Eur Rev Med Pharmacol Sci.

[42] Shahbaznejad L (2022). Epidemiological data of national Kawasaki disease registry in Iran, 2007–2019. Front Pediatr.

[43] Holman RC (2010). Racial/ethnic differences in the incidence of Kawasaki syndrome among children in Hawaii. Hawaii Med J.

[44] Lo MS (2020). A framework for understanding Kawasaki disease pathogenesis. Clin Immunol.

[45] Bahlous A (2022). Vitamin D in healthy Tunisian population: preliminary results. J Med Biochem.

[46] Lee J, Lee YJ, Kim Y (2021). A high prevalence of prediabetes and vitamin D deficiency are more closely associated in women: results of a cross-sectional study. J Int Med Res.

[47] Varghese SB, Benoit J, McIntyre T (2022). Vitamin D Levels in Ethnic Minority Adolescents in Primary Care. J Pediatr Health Care.

[48] Yin X (2022). Association between vitamin D serum levels and insulin resistance assessed by HOMA-IR among non-diabetic adults in the United States: results from NHANES 2007–2014. Front Nutr.

[49] Chakhtoura M, Napoli N, El Hajj Fuleihan G (2020). Commentary: myths and facts on vitamin D amidst the COVID-19 pandemic. Metabolism.

[50] Smolders J (2021). Letter to the Editor: Vitamin D deficiency in COVID-19: mixing up cause and consequence. Metabolism.

[51] Xie LP (2020). Epidemiologic features of Kawasaki disease in Shanghai from 2013 through 2017. J Epidemiol.

[52] Zhang D (2020). Insights into coronary artery lesions in kawasaki disease. Front Pediatr.

[53] Wang L (2021). Kawasaki disease- management strategies given symptoms overlap to COVID-19: a review. JNMA J Nepal Med Assoc.

[54] Men Q-X, Li X-M, Zhao N (2022). Influencing factos of coronary artery disease in children with Kawasaki disease. Clin Med.

[55] Stounbjerg NG (2023). Vitamin D status of 3-year-old children in Denmark: determinants and associations with bone mineralisation and blood lipids. Eur J Nutr.

[56] Makino N (2015). Descriptive epidemiology of Kawasaki disease in Japan, 2011–2012: from the results of the 22nd nationwide survey. J Epidemiol.

