# Supplementary material for: The effect of vitamin D status on the occurrence of Kawasaki Disease: a meta-analysis
Source: BMC Pediatr. 2024 Apr 29;24:287. doi: 10.1186/s12887-024-04768-1 (PMC11057174; doi:10.1186/s12887-024-04768-1)
Supplement: Supplementary file 1 — Supplementary Material 1. [file 12887_2024_4768_MOESM1_ESM.docx]

**The Effect of Vitamin D Status on the Occurrence of Kawasaki Disease: A Meta-Analysis**

**MATERIALS AND METHODS**

**Literature search**

The literature search was conducted in PubMed and Web of Science datasets for studies in English exploring the relationship between vitamin D status and KD. The search terms used were (Kawasaki disease OR Kawasaki-like syndrome OR Kawasaki syndrome OR mucoskin lymphnode syndrome) AND (vitamin D OR 25(OH)D OR 25(OH)D3 OR 25-hydroxyvitamin D3). A similar search was also conducted in China National Knowledge Infrastructure (CNKI) and Wanfang datasets using the terms “Vitamin D” and “Kawasaki Disease” for relevant reports in Chinese. Only studies published until February 2023 were included in this study, and any disagreements were resolved through discussion until consensus was reached or with the input of a third author.

**Data extraction and quality assessment**

Besides the traditional variables (*e.g.*, the first author, time of publication, sample size, vitamin D levels, and its SD or SE, ethnicity, mean age of subjects, the proportion of females) for meta-analysis extracted from eligible studies, other relative factors were also included. As the main complication of KD, the difference between patients with CAL and without CAL was often discussed (1-3), so patients with/without CAL condition were also considered by a subgroup analysis. In followed hierarchical meta-analyses, all eligible studies were categorized into three groups, they were: **CAL group** studies whose KD patients were with CAL; **NCAL group** studies whose KD patients were without CAL; studies that did not inform the CAL condition of KD patients as **mix group**. Although, there are more than eight different scoring methods (*e.g.*, the Harada score, Egami score, Kobayashi score, Tang score, Hua score, *etc.*) are employed in delineating the severity of KD and/or predicting efficacy of IVIG or GAL, the traditional KD diagnostic criteria were used in the most of eligible studies in current meta-analysis.

Among included studies, there were different units (*e.g.*, ng/mL; nmol/L; mg/dL, *etc.*) were used to measure the levels of vitamin D in serum (Table S1). All these data will be converted and unified with the unit of ng/mL.

In the eligible studies, various versions of KD diagnosis and CAL diagnosis criteria were applied, including the 2004 and 2017 versions proposed by the American Heart Association (AHA2004 and AHA2017; 5, 6), different versions proposed by the Japanese Association for Infectious Disease/Japanese Society of Chemotherapy (JAID/JSC 5^th^ and JAID/JSC 6^th^; 7) , and versions proposed by the Committee of the Chinese Journal of Pediatrics (8) (**Table S1**). However, due to the multitude of versions and insufficient current research data, we lack the support to analyze the potential impact of different KD diagnostic criteria on the results.

There is significant variation in the methods used to measure individual vitamin D levels in these studies. While most studies measure 25(OH)D using the ELISA method, the specific detection equipment and reagents used vary. Additionally, some studies utilize chemiluminescence of electrochemiluminescence techniques (**Table S1**). However, the potential impact of these factors on our analysis results cannot be accurately assessed.

The quality of all included studies was also evaluated with the Newcastle-Ottawa Scale (NOS) assessment scale (4). All these works, including literature search, data extraction, and unification have been done by two independent authors (H.Z and J.W).

**Statistical analysis**

*Outlier detection and re-analysis* Studies with extreme effect sizes, known as outliers, may distort the overall results of a meta-analysis (9). To address this issue, the *influence.analysis* R program was utilized to detect and remove outlier (s) among all eligible studies using various influence measures (*e.g.*, DIFFITS, Cook’s distance, covariance ratio, *etc.*). After removing the outlier(s), the meta-analysis was conducted again.

*Sensitivity and publication bias analysis* To account for the significant heterogeneity (*P*_het_ ≤ 0.05 or *I* ^2^ ≥ 25%) observed, sources of heterogeneity were considered (10). *Begg’s* rank correlation test and *Egger’s* weighted regression test were used to detect publication bias. The results of the “*trim-and-fill*” test were visualized through a funnel plot. The leave-one-out function examined the sensitivity of the results. To maintain the Type I error rate, the “*permutest*” command of the *Metafor* package was utilized to conduct a permutation test with 1,000 iterations.

*Meta-regression analysis* To investigate the potential causes of observed heterogeneity and determine their impact on the correlation between vitamin D and KD, a meta-regression analysis was conducted using a mixed-effects model, which utilized a random-effects model within subgroups and a fixed-effects model among subgroups (11). The model test (*Q*_M_) which is a simultaneous test that all variables (except the intercept) are zero, was used to test whether at least one of the variables is related to the effect size. The Goodness of fit test (*Q*_M_) was also used to test whether is there any unexplained variance in the true effect size (9). Five types of information were examined in the meta-regression analysis: publication date, the mean age of participants, the percentage of females, race, and presence of KD complications, all of which were considered as possible covariates.

**RESULTS**

**1.1 Outlier(s) detection**

To minimize the outlier(s) influence, the program of *influence-analysis* in R was used for the detection and removal of outlier(s) from total studies. Because that outlier usually has a decreased quality and extreme effect size, and is possible distorted the overall estimate. As described in **Figure S1**, one study was identified as an outlier among these eligible studies.

**1.2 Sensitivity analysis**

In order to obtain a robust estimate of serum 25(OH)D on KD, we conducted a sensitivity analysis. In each meta-analysis, only a single study removed was deleted to obtain the estimated values of its remaining studies (there will be 22 - 1 = 22 results). Then, by comparing the deviation from the range of 95%CI of overall effect size, we can determine whether there were single or multiple samples that will interfere with our overall analysis results. **Figure S2** indicated that these effect sizes (from -1.46 to -1.14 ng/mL) did not significantly deviate from the range of the overall effect’s 95%CI interval (-1.72 to -0.15 ng/mL) when removing any single study. The sensitivity analysis indicated that the result is sufficiently robust.

**1.3 Meta-regression analysis**

In our meta-regression analysis, we considered five variables - age, gender, publication time, race and complication of KD patients – to investigate the potential reasons for the significant heterogeneity observed in our primary meta-analysis (Cochran’s *Q* = 1,206, *df* = 21; and *I*^2^ = 98.26; *P* < 0.001). The result of the meta-regression analysis indicated that these variables did not significantly contribute to such extreme heterogeneity (**Table S1**). Nevertheless, further assessments of predictive effect of individual vitamin D levels in combination with these five variables and a comprehensive test of this model produced more valuable information. The result of the test of model (*Q*_M_ = 2.89, *df* = 6, *P* = 0.82) also accepted the null hypothesis and concluded that none of the covariates (among five included variables) significantly impact the relationship between vitamin D and KD. The Goodness of fit test demonstrated significant deviation of each study from its predicted values (*Q*_G_ = 834.96, *df* = 12, *P* < 0.001), suggesting that the current model was incomplete. In other words, knowing a study’s five variables as mentioned above could not allow us to entirely prediction the effect of vitamin D on KD.

**References**

1. Chen X, Luo X, Ma X, Wong J, Wu H and Zhang Y (2019). "Serum 25 hydroxyvitamin D3 in predicting coronary arterial lesions of Kawasaki Disease." Chinese Journal of Woman and Child Health Research 30(5): 558-561.
2. Zhang X, Li C, OU Z, Lin N and Zheng X (2019). "Changes and clinical significance of serum 25-hydroxyvitamin D levels in children with Kawasaki disease." China Modern Medicine 26(5): 98-100.
3. Jing H, Gao J and Han W (2020). "Study on serum vitamin D level and vitamin D receptor Fok I gene polymorphism in children with Kawasaki disease." Maternal and Child Health Care of China 35(3): 459-462.
4. Peterson J, Welch V, Losos M and Tugwell P (2014). The Newcastle-Ottawa Scale (NOS) for Assessing the Quality of Non-Randomised Studies in Meta-Analyses. Ottawa: Ottawa Hospital Research Institute, 2(1), 1-12.
5. McCrindle B, Rowley A, et al (2017). Diagnosis, Treatment, and Long-Term Management of Kawasaki Disease: A Scientific Statement for Health Professionals From the American Heart Association. Circulation 135(17): e927-e999.
6. Newburger JW, Takahashi M, et al (2004). Diagnosis, Treatment, and Long-Term Management of Kawasaki Disease: a statement for health professionals from the Committee on Rheumatic Fever, Endocarditis and Kawasaki Disease, Council on Cardiovascular Disease in the Young, American Heart Association. Circulation 110:22747-2771.
7. Japanese Association for Infectious Disease/Japanese Society of Chemot, J. J. G. G. t. C. M. o. I. D. P. Committee, et al (2017). JAID/JSC Guidelines for Clinical Management of Infectious Disease 2015 - Urinary tract infection/male genital infection. Journal of infection and chemotherapy: official journal of the Japan Society of Chemotherapy 23:733-751.
8. Editorial Committee of Chinese Journal of Pediatrics, Immunology Group of Chinese pediatric Society (2007). Summary of Kawasaki Diseasee Symposium. Chinese Journal of Pediatrics 45: 826-830.
9. Viechtbauer W (2010). "Conducting meta-analyses in R with the *metafor* package." Journal of statistical software 36(3): 1-48.
10. Higgins J, and Thompson S (2002). "Quantifying heterogeneity in a meta-analysis." Stat Med 21(11): 1539-1558.
11. Harrer M, and Ebert D (2018). Doing Meta-Analysis in R: A Hand-on Guide. Boca Raton, FL and London: Chapman & Hall/CRC Press. ISBN 978-0-367-61007-4.

**Figures**


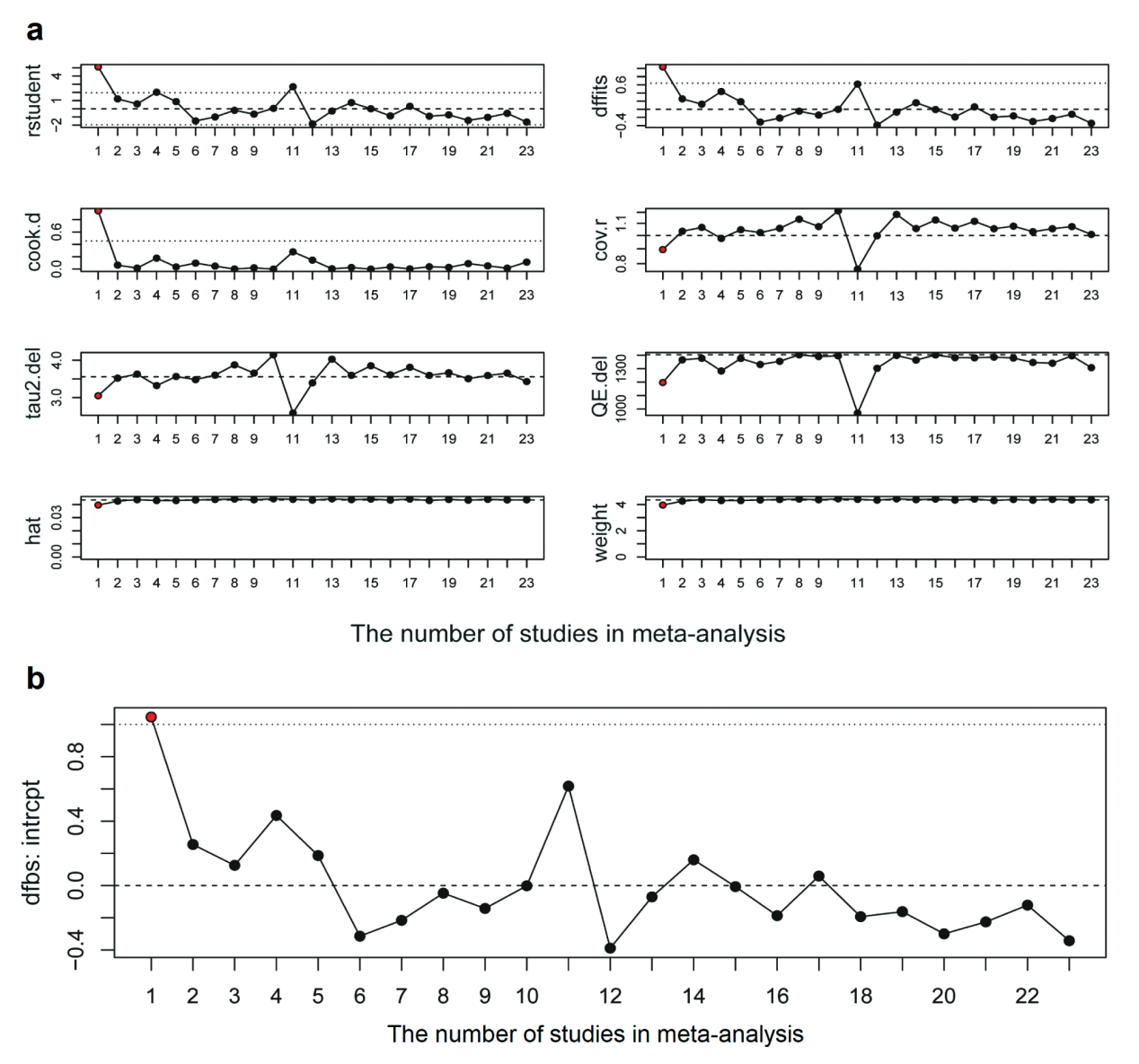


**Figure S1** Plot of the various diagnostic measures of influence analyses for relationship between serum 25(OH)D levels on KD. The influence of each study were measured with: a) **retudent**, the externally standardized residuals; **diffits**, DFFITS values; **cook.d**, Cook’s distances; **cov.r**, covariance ratios; **tau2.del**, estimates of T2; **QE.del**, the test statistics for (residual) heterogeneity when each study is removed in turn; hat, the diagonal elements of the hat matrix; **weight**, the weights (in%) given to observed outcomes during the model fitting; and b) **DFBETAS** values. Study considered to be influential, was colored in red in the plot.


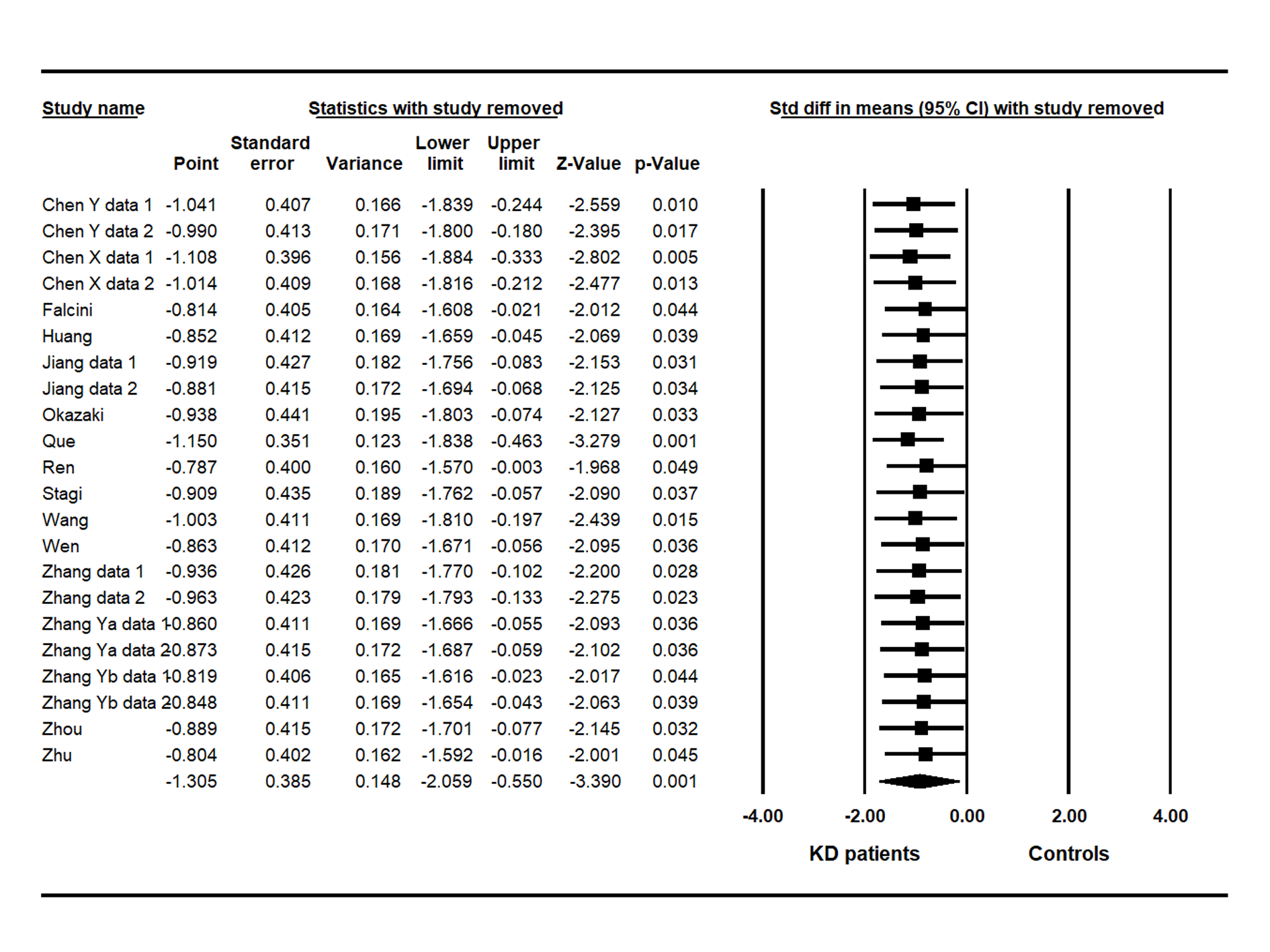


**Figure S2** The sensitivity analysis of the effect size of 25(OH)D on KD by leave-one-out method

**Table S1.** The related information of included studies.

| Author, year | Country | N (case/ control) | age | M% | Measurement for 25(OH)D | Diagnostic criteria | Units |
| --- | --- | --- | --- | --- | --- | --- | --- |
| An 2016 | Chinese | 45/43 | 3.1 | 53.5 | ELISA (Roche, Switzerland) | JAID/JSC 5^th^ | ng/mL |
| Chen 2014 | Chinese | 9/30 | n.a. | 64.6 | ELISA (Xinle, China) | AHA2004 | ng/mL |
| Chen 2014 | Chinese | 26/30 | n.a. | 64.6 | ELISA (Xinle, China) | AHA2004 | ng/mL |
| Chen 2019 | Chinese | 30/30 | 3.3 | 57.7 | ELISA (WaLan, China) | JAID/JSC 5^th^ | ng/mL |
| Chen 2019 | Chinese | 11/30 | 3.3 | 57.7 | ELISA (WaLan, China) | JAID/JSC 5^th^ | ng/mL |
| Falcini 2015 | Italian | 60/60 | 1.98 | 70 | n.a | n.a | mg/dL |
| Huang 2022 | Chinese | 105/45 | 2.9 | 53.3 | Electrochemiluminescence technique (Roche, Cohas 601) | AHA2017 | ng/mL |
| Jiang 2020 | Chinese | 74/80 | 2.18 | 54.1 | ELISA (JiangLai, China) | AHA2004 | ng/mL |
| Jiang 2020 | Chinese | 4/80 | 2.3 | 62.5 | ELISA (JiangLai, China) | AHA2004 | ng/mL |
| Okazaki | Japan | 86/290 | 2.1 | 57.4 | Radioimmunoassay (DiaSorin, Italy) | AHA2004 | ng/mL |
| Que 2022 | Chinese | 98/96 | 1.15 | 68.6 | Electrochemiluminescence technique (Beckman, US) | n.a | ng/mL |
| Ren 2021 | Chinese | 66/66 | 3.46 | 62.9 | ELISA (Beckman, US) | JAID/JSC 6^th^ | ng/mL |
| Stagi 2016 | Italian | 79/234 | 4.7 | 66.1 | ELISA (Beckman, US) | AHA2004 | ng/mL |
| Wang 2015 | Chinese | 35/25 | n.a | 74.3 | ELISA (n.a) | n.a | ng/mL |
| Wen 2018 | Chinese | 50/30 | 2.9 | 52.5 | ELISA (n.a) | n.a | ng/mL |
| Zhang 2019 | Chinese | 76/58 | 1.8 | 60 | ELISA (Rocha, Switzerland) | AHA2017 | nmol/L |
| Zhang 2019 | Chinese | 71/58 | 1.8 | 60 | ELISA (Rocha, Switzerland) | AHA2017 | nmol/L |
| Zhang 2018 | Chinese | 20/45 | 3.2 | 55.9 | ELISA (BuoHui, China) | AHA2004 | ng/mL |
| Zhang 2018 | Chinese | 75/45 | 3.2 | 55.9 | ELISA (BuoHui, China) | AHA2004 | ng/mL |
| Zhang 2016 | Chinese | 63/40 | 3.2 | 61.9 | ELISA (BuoHui. China) | AHA2004 | ng/mL |
| Zhang 2016 | Chinese | 179/40 | 3.4 | 55.3 | ELISA (BuoHui, China) | AHA2004 | ng/mL |
| Zhou 2022 | Chinese | 40/30 | 2.4 | 38.4 | Chemiluminescence technique (n.a) | JAID/JSC 5^th^ | nmol/L |
| Zhu 2021 | Chinese | 72/72 | 3.42 | 57.6 | Electrochemiluminescence technique (n.a) | AHA2004, JAID/JSC 5^th^ | ng/mL |

Abbreviation: N, the number of participants (cases and controls) in each study; M%, percentage value of male in the sample; ELISA, the enzyme-linked immunosorbent assay; 25(OH)D, 25-hydroxyvitamin D; JAID/JSC, the guidelines (5^th^ and 6^th^ versions) proposed by the Japanese Association for Infectious Disease/ Japanese Society of Chemotherapy; AHA, the guidelines (2004, and 2017 versions) proposed by the American Heart Association; n.a, not available.

**Table S2** Meta-regression analysis for relationship between vitamin D status and KD under random-effect model.

| Covariate | *Coefficient (β)* | *SE* | 95%CI | | *z* | *P* |
| --- | --- | --- | --- | --- | --- | --- |
|  |  |  | *Lower* | *Upper* |  |  |
| Intercept | -708.83 | 646.64 | -1976.21 | 558.56 | -1.10 | 0.27 |
| Publication time | 0.35 | 0.32 | -0.28 | 0.97 | 1.09 | 0.27 |
| Sex | 5.24 | 8.50 | -11.43 | 21.91 | 0.62 | 0.54 |
| Age | -0.34 | 0.68 | -1.68 | 1.01 | -0.49 | 0.62 |
| Complication ^a^ | *Q* = 0.69, *df* = 2, *P* = 0.71 | | | | | |
| CAL | 1.08 | 1.50 | -1.87 | 4.03 | 0.72 | 0.47 |
| NCAL | 1.14 | 1.50 | -1.79 | 4.08 | 0.76 | 0.44 |
| Race: Asian ^b^ | -0.54 | 1.84 | -4.15 | 3.06 | -0.30 | 0.77 |

Abbreviation: KD, Kawasaki disease; CAL, KD without coronary artery lesions; NCAL, KD without coronary artery lesions; Sex, the proportion of female in each sample; Age, the mean of age in each sample. Test of model: *Q*_M_ = 2.89, *df* = 6, *P* = 0.82; Goodness of fit test: *Q*_G_ = 834.96, *df* = 12, *P* < 0.001. a. samples including patients with/without CAL (mix) as reference. b, samples of Caucasian as reference.
